# PPARs in Neuroinflammation

**DOI:** 10.1155/2008/638356

**Published:** 2008-08-03

**Authors:** Michael K. Racke, Paul D. Drew

**Affiliations:** ^1^Department of Neurology, The Ohio State University Medical Center, 1654 Upham Drive, 445 Means Hall, Columbus, OH 43210-1228, USA; ^2^Department of Neurobiology and Developmental Sciences, University of Arkansas for Medical Sciences, Slot 846, 4301 W. Markham Street, Little Rock, AR 72205, USA

Welcome to this special issue of PPAR Research dedicated to “PPARs in Neuroinflammation.” The central nervous system (CNS) was once thought to be an immune-privileged site void of significant inflammation. However, it is now clear that activated peripheral immune cells are capable or entering and functioning within the CNS. In addition, resident immune cells termed “microglia” protect the CNS through production of molecules which are toxic to pathogens. However, chronically activated microglia can produce molecules toxic to host CNS cells, potentially leading to neurodegeneration. Interestingly, a variety of CNS disorders are characterized by neuroinflammation and associated neurodegeneration. The role of PPARs in modulating lipid and glucose metabolism is well established. More recently, PPARs have been demonstrated to modulate inflammation. For example, PPAR agonists inhibit the production of proinflammatory molecules by peripheral immune cells as well as resident CNS glia. Furthermore, PPAR receptor agonists have proven effective in suppressing the development of animal models of CNS inflammatory and neurodegenerative disorders. This suggests that modulation of PPARs may be effective in treating the related human diseases.

This special issue of PPAR Research contains a series of reviews concerning the role of PPARs in neuroinflammatory diseases. We are fortunate to have received contributions from experts in the fields concerning the potential role of PPARs in modulating CNS disorders including multiple sclerosis, Alzheimer’s disease, spinal cord injury, stroke, traumatic brain injury, amyotrophic lateral sclerosis, and Huntington’s disease. Also included are reviews concerning the role of PPAR agonists in modulating the function of resident CNS microglia, and the molecular mechanisms by which PPARs regulate inflammatory signaling as related to CNS disease.

We are pleased that this special issue of PPAR Research also contains two original research reports. The first report provides a thorough investigation of the effects of PPAR-γ agonists in modulating the production of proinflammatory molecules by CNS microglia and astrocytes in response to distinct toll-like receptor ligands relevant to infections of the CNS. The second report investigates PPAR-γ agonist effects on amyloid beta-mediated microglial production of cytokines known to alter T-cell differentiation. This study may have important implications concerning the use of amyloid beta immunization for the treatment of Alzheimer’s disease.

We hope that you find this special issue of PPAR Research dedicated to PPARs in neuroinflammation to be informative, and that the special issue will generate additional interest in this rapidly evolving field of research.

*Michael K. Racke* *Michael K. Racke* *Paul D. Drew* *Paul D. Drew*

